# Mesenchymal Stem Cell-Derived Exosomes in Skin Wound Healing and Scar Prevention: Mechanisms, Comparison, and Clinical Prospects

**DOI:** 10.1007/s13770-026-00821-5

**Published:** 2026-07-17

**Authors:** Yaoyao Song, Fei Xie, Xiangdang Liang

**Affiliations:** 1 Senior Department of Burns and Plastic Surgery, Fourth Medical Center of Chinese PLA General Hospital, Beijing, China; 2 Senior Department of Orthopedics, Fourth Medical Center of Chinese PLA General Hospital, No. 28, Fuxing Road, Beijing, 100853 People’s Republic of China

**Keywords:** Mesenchymal stem cell-derived exosomes, Wound healing, Scar prevention

## Abstract

**Background:**

Exosomes derived from mesenchymal stem cells (MSCs) have garnered significant research interest for their roles in promoting wound healing and preventing scar formation. Studies have shown that MSC-derived exosomes (MSC-Exos) accelerate the repair of cutaneous and corneal wounds through multiple mechanisms, including modulation of cell migration, anti-inflammatory responses, inhibition of apoptosis, collagen remodeling, and promotion of angiogenesis. Notably, exosomal miRNAs play a pivotal role in inhibiting fibroblast-to-myofibroblast transformation and modulating the TGF-β signaling pathway, thereby effectively reducing scar formation. Promising outcomes of exosomes derived from adipose tissue- and umbilical cord blood-derived MSCs in various wound models underscore their clinical potential.

**Methods:**

This review systematically elaborates on the specific mechanisms by which MSC-Exos promote wound healing and inhibit scar formation, and summarizes their application outcomes in different wound models. Additionally, it explores current advanced biomaterial delivery systems designed to optimize exosome delivery, aiming to provide insights and future directions for the clinical translation of MSC-Exos.

**Results and conclusion:**

MSC-Exos effectively promote tissue repair and reduce scarring through multi-target and multi-pathway mechanisms, demonstrating significant clinical application prospects. Delivery systems incorporating biomaterials hold promise for further enhancing their therapeutic efficacy and stability. Future research should focus on elucidating the molecular mechanisms of exosome action, developing scalable preparation protocols, and conducting preclinical and clinical evaluations to advance their translation into clinical therapeutic applications.

## Introduction

As the body's largest organ, skin is frequently subjected to injury. While the wound healing process is a well-orchestrated sequence of events—hemostasis, inflammation, proliferation, and remodeling—its dysregulation can lead to chronic wounds or pathological scarring, imposing significant burdens on patients and healthcare systems. Current therapies often fail to achieve both rapid healing and aesthetically acceptable, functional scar outcomes, highlighting the need for innovative regenerative approaches [1, 2].

In recent years, mesenchymal stem cell-derived exosomes (MSC-Exos) have emerged as a forefront cell-free therapeutic paradigm. These nano-sized extracellular vesicles serve as natural carriers of bioactive molecules (proteins, lipids, miRNAs, etc.), mediating intercellular communication. They recapitulate many therapeutic functions of their parent MSCs—such as modulating inflammation, promoting angiogenesis, and stimulating tissue repair—while offering advantages like lower immunogenicity and easier storage [3]. Critically, emerging evidence positions MSC-Exos not merely as accelerators of healing but as key regulators capable of steering the repair process towards a regenerative, scar-minimized outcome by fine-tuning the behavior of fibroblasts, immune cells, and endothelial cells within the wound microenvironment [4, 5].

This comprehensive review aims to provide a focused and critical analysis of the current landscape of MSC-derived exosomes in the context of skin wound healing and scar prevention. First, we will systematically elaborate on the multifaceted mechanisms by which MSC-derived exosomes promote wound healing and inhibit scar formation. Subsequently, we will compare the functional characteristics and therapeutic distinctions of exosomes derived from different tissue sources, including bone marrow, adipose tissue, umbilical cord blood, and umbilical cord. Recognizing the challenges of direct application, we will explore targeted delivery and functional enhancement strategies based on advanced biomaterials, and critically discuss the pressing challenges in clinical translation. By integrating mechanistic insights with translational perspectives, this review seeks to identify key knowledge gaps and future research directions essential for fully harnessing the potential of MSC-derived exosomes in transformative dermatological therapies.

## Overview of cutaneous wound healing and scar formation

Cutaneous wound healing is a dynamic and continuous process, traditionally divided into four overlapping phases: hemostasis, inflammation, proliferation, and remodeling. The hemostatic phase achieves initial wound closure via fibrin clot formation; the inflammatory phase is characterized by the recruitment of neutrophils and macrophages, which clear tissue debris and pathogens; the proliferative phase relies on the coordinated action of keratinocytes, fibroblasts, and endothelial cells to drive re-epithelialization, granulation tissue formation, and angiogenesis; finally, during the remodeling phase, the extracellular matrix undergoes reorganization and collagen progressively matures, restoring tissue structure and functional integrity [6].

Pathological scar formation often stems from aberrations in the remodeling process, with its core pathological mechanism involving the persistent activation and survival of myofibroblasts. In normal healing, myofibroblasts appear transiently to mediate wound contraction and are subsequently cleared via apoptosis. In pathological scars, such as hypertrophic scars and keloids, sustained inflammatory stimuli and dysregulated signaling pathways like TGF-β inhibit myofibroblast apoptosis, leading to excessive deposition of extracellular matrix, particularly an imbalance in the ratio of type I to type III collagen. This aberrant metabolism results in disorganized, thickened collagen fibers, forming fibrotic scar tissue [7, 8]. Therefore, targeting the differentiation, function, and survival of myofibroblasts, while correcting the imbalance between collagen synthesis and degradation, has become a critical strategic direction for preventing and treating pathological scars.

**Fig. 1:**
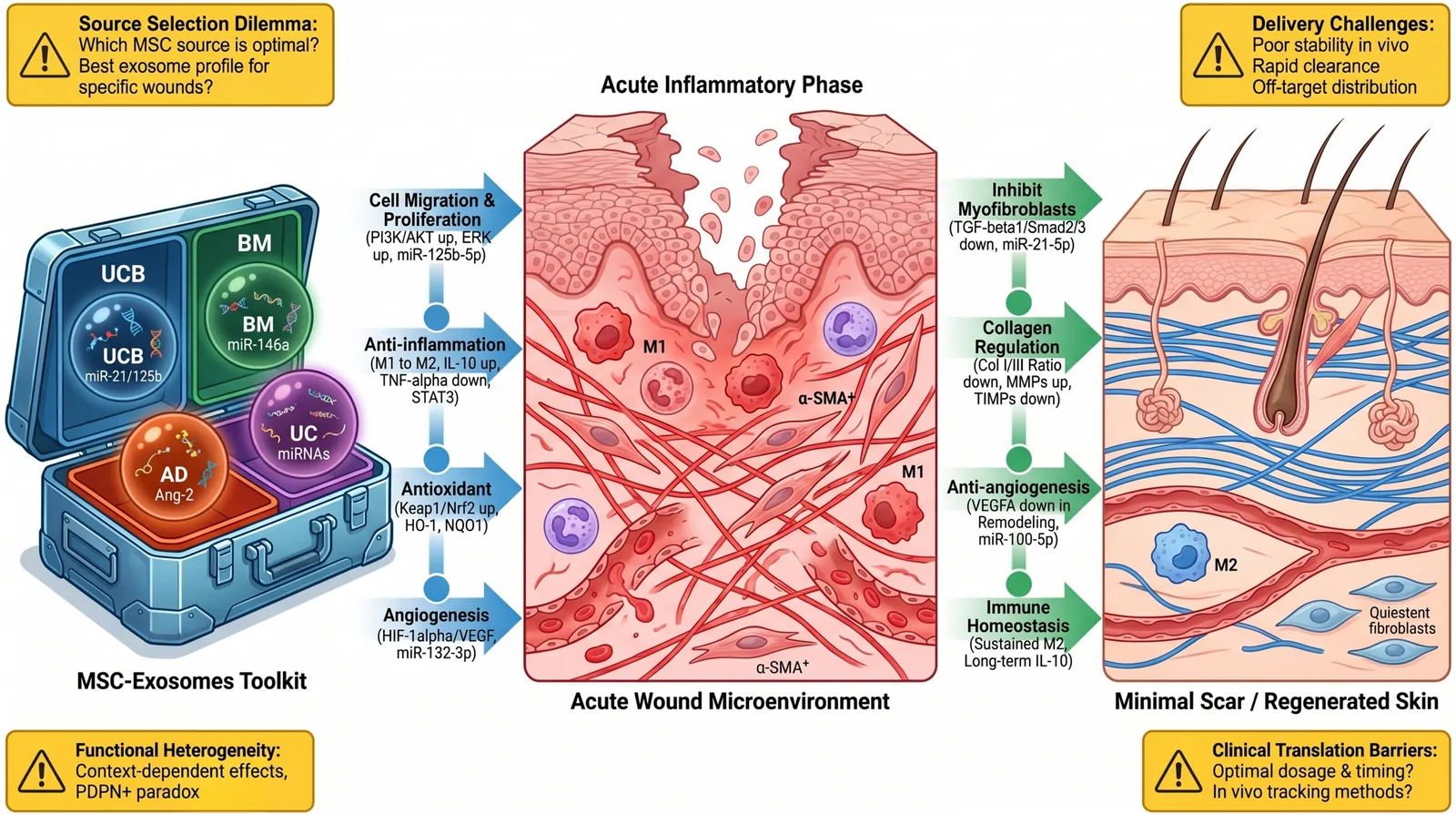
Schematic overview of exosome biogenesis and release

In summary, cutaneous wound healing and scar formation is a complex, multi-stage, and precisely regulated yet easily dysregulated process (Fig. 1). The following section will elaborate on how MSC-derived exosomes intervene in this process.

## Mechanisms of MSC-exosomes in wound healing and scar prevention

### Mechanisms by which MSC-derived exosomes promote wound healing

#### Promoting cell migration and proliferation

MSC-Exos promote the migration and proliferation of wound-related cells (e.g., keratinocytes, fibroblasts) by carrying bioactive molecules that activate key signaling pathways like PI3K/Akt and ERK. This is a fundamental mechanism for accelerating wound closure. The cargos and target cell preferences can vary by source. For instance, bone marrow MSC-derived exosomes (BMSC-Exos) promote the migration of brain microvascular endothelial and tendon stem/progenitor cells, demonstrating a general pro-migratory capacity relevant to skin repair [9, 10]. Adipose-derived MSC exosomes (ADSC-Exos) activate the AKT/HIF-1α axis to promote keratinocyte proliferation and migration [11, 12]. Umbilical cord Wharton’s jelly-derived MSC-Exos(UC-Exos), rich in fibrinogen, effectively promote keratinocyte growth and migration[13]. In summary, MSC-Exos provide a solid cellular foundation for wound healing by enhancing cell motility and growth through multiple pathways.

#### Anti-inflammatory and immunomodulatory effects

MSC-Exos play a crucial anti-inflammatory and immunomodulatory role in the wound healing process, achieved by regulating the local inflammatory microenvironment, inhibiting the release of pro-inflammatory cytokines, and promoting the transition of inflammation from the pro-inflammatory phase to the repair phase. Multiple studies have shown that MSC-Exos can modulate the activity of immune cells, reducing the negative impact of chronic inflammation on wound healing, thereby promoting tissue regeneration and functional recovery.

A key mechanism involves promoting the polarization of macrophages toward the anti-inflammatory, pro-repair M2 phenotype while inhibiting the pro-inflammatory M1 phenotype. This shift reduces the secretion of factors such as TNF-α and IL-1β and enhances the release of anti-inflammatory mediators like IL-10 [14, 15]. For example, BMSC-Exos induce M2 macrophage polarization both in vitro and in vivo, suppressing the expression of pro-inflammatory factors and enhancing the release of anti-inflammatory factors, thereby alleviating neuroinflammation and promoting tissue repair [16]. ADSC-derived exosomes exhibit anti-inflammatory effects in inflammatory bowel disease models [17], and UC-Exos can reverse the pro-inflammatory state of osteoarthritis chondrocytes [18]. The immunomodulatory functions are mediated by exosomal miRNAs (e.g., miR-16, miR-21) and proteins (e.g., galectin), which regulate pathways such as IL-10/STAT3 and NF-κB.

While the anti-inflammatory prowess of MSC-Exos is well-documented, its translation to predictable scar prevention requires caution. Most evidence comes from in vitro models or acute injury models (e.g., neural, osteoarthritic). The chronic, dysregulated inflammation in human hypertrophic scars or keloids presents a more complex microenvironment. Furthermore, the cited study on post-myocardial infarction fibrosis reveals a paradoxical scenario where a specific MSC subpopulation’s exosomes (PDPN +) can exacerbate fibrosis via SAA3-amyloid deposition [19]. This underscores a critical principle: the immunomodulatory output of MSC-Exos is highly context-dependent, influenced by the parent cell’s state and the disease milieu. Assuming a uniformly anti-inflammatory effect in all wound scenarios is an oversimplification.

#### Antioxidant and anti-apoptotic functions

MSC-Exos protect tissues from oxidative damage and cell death. ADSC-Exos can regulate the Bax/Bcl2 ratio to inhibit apoptosis, protecting corneal cells and preventing α-SMA expression [20]. The antioxidant effect is often mediated through the Keap1/Nrf2 pathway. BMSC-Exos activate this pathway, upregulating antioxidant enzymes like SOD and GPx, and reducing ROS levels. Similarly, miR-200a-3p in UC-Exos targets Keap1 to activate Nrf2, alleviating UVB-induced skin oxidative damage [21, 22]. The anti-apoptotic effect is also achieved through miRNAs; BMSC-Exos carry miR-24-3p, which targets MAPK9 to inhibit the JNK/c-Jun/c-Fos pathway, reducing apoptosis and promoting neural repair [23].

The antioxidant and anti-apoptotic effects of MSC-Exos are compelling, but the evidence base is largely built on studies from non-skin organ systems (e.g., kidney [24], ovary [25], spinal cord [23]). While pathways like Keap1/Nrf2 are universally important, cell-type-specific responses and the unique oxidative stress landscape of the skin necessitate direct validation in dermal cells and relevant skin injury models. Future research must quantify the direct impact of exosome-mediated oxidative stress reduction on human dermal fibroblast activation and collagen metabolism.

#### Promoting angiogenesis and tissue remodeling

Adequate vascularization is vital for wound healing. MSC-Exos promote angiogenesis by delivering pro-angiogenic factors.

ADSC-Exos carry miR-132 and miR-146a to upregulate pathways promoting human umbilical vein endothelial cell (HUVEC) proliferation and tube formation [26]. Hypoxia-preconditioned UC-MSC-Exos transport miR-205-5p to activate the PTEN/PI3K/AKT/mTOR pathway, enhancing endothelial function [27]. BMSC-Exos in hyaluronic acid hydrogels promote bone regeneration via the miR-21-mediated NOTCH1/DLL4 pathway, highlighting a role in vascularized tissue repair [28]. UC-MSC-Exos deliver Angiopoietin-2, directly promoting HUVEC proliferation, migration, and tube formation [26]. Engineered modifications, like overexpression of HIF-1α in MSC-Exos, further enhance angiogenic potential [29, 30].

Meanwhile, MSC-derived exosomes also play a crucial role in promoting wound structural remodeling and reducing the risk of fibrosis. They regulate collagen deposition and alignment, facilitating the orderly reconstruction of the extracellular matrix, thereby mitigating excessive fibrosis and scar formation. For example, UC-MSC-derived exosomes not only promote angiogenesis through the delivery of angiopoietin-2 to accelerate healing, but the variety of active molecules they carry (such as specific miRNAs and lncRNAs) can also form a regulatory network [31]. This network synergistically promotes functional tissue remodeling by inhibiting inflammation, modulating the immune microenvironment, and enhancing the proliferation and migration of fibroblasts and keratinocytes [32, 33]. These effects collectively improve the wound microenvironment and support tissue regeneration.

### The role and molecular mechanisms of MSC exosomes in preventing scar formation

#### Inhibiting myofibroblast differentiation

The transformation of fibroblasts into α-smooth muscle actin (α-SMA)-positive myofibroblasts is a cornerstone of scar formation, primarily driven by TGF-β signaling. MSC-Exos can inhibit this process. A prime example is umbilical cord blood MSC-Exos (UCB–Exos), which are rich in miR-21-5p and miR-125b-5p. These miRNAs directly target and inhibit TGF-β receptor II (TGFBR2) and receptor I (TGFBR1), respectively, effectively blocking the TGF-β1/Smad2/3 signaling pathway, reducing α-SMA and collagen I/III expression, and inhibiting myofibroblast differentiation [34, 35]. Similarly, ADSC-Exos contain miR-181a-5p that targets Smad2 mRNA, regulating the TGF-β pathway and reducing myofibroblast transdifferentiation [36]. BMSC-Exos and 3D-cultured UC-Exos (carrying let-7i-5p) also show efficacy in inhibiting fibroblast activation and alleviating fibrosis in various models [37, 38].

Collectively, these findings underscore that inhibition of the TGF-β/Smad pathway is a central mechanism by which MSC-Exos exert anti-fibrotic effects, as exemplified by the direct miRNA-mediated targeting of TGF-β receptors in the case of UCB-Exos [39]. While this provides a strong molecular rationale, several critical gaps in our current understanding remain. First, the comparative efficacy of exosomes from different sources is not well established, it is unclear whether they inhibit myofibroblast differentiation with equal potency or through entirely distinct miRNA cargo profiles. Second, the translational relevance of these findings may be limited, as most supporting evidence comes from in vitromodels that rely on TGF-β1 stimulation—a simplification that may not fully replicate the complex and sustained pro-fibrotic signaling network present in a mature scar in vivo. Addressing these gaps is essential to accurately evaluate and harness the therapeutic potential of MSC-Exos in clinical scar prevention.

#### Regulating collagen synthesis and remodeling

Scar tissue is characterized by disordered and excessive collagen deposition, particularly an increased Type I/III collagen ratio. MSC-Exos can modulate this balance. ADSC-Exos inhibit TGF-β1-induced over-synthesis of collagen I and III while upregulating matrix metalloproteinases (MMP1, MMP3) to promote collagen degradation. In anterior cruciate ligament repair, BMSC-Exos promoted Type I collagen synthesis while relatively suppressing Type III collagen, leading to more organized fiber formation and improved mechanical properties [40, 41]. This regulation involves miRNAs like miR-181a and signaling pathways such as p38 MAPK and PI3K/Akt [42–44]. By promoting a more balanced collagen metabolism and orderly deposition, MSC-Exos help improve the quality of healed tissue and reduce scarring.

#### Anti-angiogenic effects

While angiogenesis is essential early in healing, excessive or persistent vascularization during remodeling can contribute to scar hyperplasia. MSC-Exos demonstrate context-dependent, biphasic regulation of angiogenesis. In the remodeling phase, they can exert anti-angiogenic effects to prevent scar formation [45]. For instance, exosomes from stem cells of human exfoliated deciduous teeth (SHED-Exos) carry miR-100-5p and miR-1246 to downregulate VEGFA, inhibiting endothelial cell proliferation and tube formation [46]. BMSC-Exos carrying ANGPTL1 inhibit angiogenesis in glioblastoma by blocking the VEGFA/VEGFR2/Akt/eNOS pathway [47]. Specific miRNAs in MSC-Exos, such as miR-185-5p, also inhibit pathological angiogenesis in models like diabetic retinopathy. This ability to switch from pro-angiogenic to anti-angiogenic modes underscores their role as fine-tuners of the healing process [48].

Furthermore, MSC-Exos are also enriched with a series of key miRNAs that promote physiological angiogenesis, and these molecules play a crucial role in the early stages of wound healing. For example, miR-21-5p can enhance endothelial cell proliferation and migration by inhibiting TGF-β1, thereby promoting angiogenesis [49]. miR-125a-3p promotes endothelial cell survival, migration, and tube formation by targeting and inhibiting PTEN [50]. In a diabetic wound healing model, miR-132-3p has been shown to promote neovascularization and accelerate healing by regulating multiple angiogenesis-related factors [51]. These specific miRNAs constitute the core molecular basis of the pro-angiogenic activity of MSC-Exos. Together with the anti-angiogenic miRNAs (such as miR-100-5p and miR-1246) they carry in specific contexts, they achieve spatiotemporally precise regulation of the wound vascularization process.

#### Contributions of anti-inflammatory and immunomodulatory effects to scar prevention

As previously mentioned, the anti-inflammatory effects directly contribute to scar prevention by resolving the chronic inflammation that drives fibrosis. MSC-derived exosomes regulate immune cells, particularly by shifting macrophages from the pro-fibrotic M1 phenotype to the anti-fibrotic M2 phenotype, thereby reducing the release of fibrotic mediators [52–54]. Importantly, the immunomodulatory function is not universally beneficial. A study on myocardial infarction scar formation revealed that a specific PDPN + MSC subpopulation secreted exosomes rich in Serum Amyloid A3 (SAA3). These exosomes activated TLR2 on macrophages, leading to excessive SAA3 production and its aggregation into rigid amyloid fibers in the ECM, exacerbating scar stiffness and impairing cardiac function [19]. This finding is a critical cautionary note, demonstrating that under specific conditions, MSC-Exos can promote, rather than inhibit, pathological fibrosis. It highlights the essential "context-dependency" of exosome function, a crucial consideration for clinical application.

### Comparison of MSC exosomes from different sources

#### Umbilical cord blood MSC exosomes (UCB- Exos)

Exosomes derived from umbilical cord blood mesenchymal stem cells exhibit significant therapeutic potential in promoting skin wound healing and inhibiting scar formation. Their core advantage lies in providing the most direct and potent anti-scar evidence through specific targeting of the transforming growth factor-β pathway [34]. UCB- Exos are enriched in miR-21-5p and miR-125b-5p. These two miRNAs directly target and inhibit TGF-β receptor II and receptor I, respectively, thereby effectively blocking the TGF-β1/Smad2/3 signaling pathway. This mechanism has been confirmed in both in vitro and in vivo experiments, demonstrating significant inhibition of the transformation of human skin fibroblasts into myofibroblasts, reduction of excessive collagen deposition in scar tissue, and promotion of skin cell proliferation and migration, ultimately achieving rapid and high-quality wound repair [34]. This clear miRNA-mediated signaling regulatory mechanism endows UCB-Exos with unique advantages in preventing scar formation, providing new insights for clinical applications [34, 55].

#### Bone marrow-derived MSC exosomes (BMSC-Exos)

BMSC-Exos are among the earliest studied sources, demonstrating multifaceted biological activities in promoting tissue repair. In wound healing, BMSC-Exos function through various mechanisms: including activating the Keap1/Nrf2 pathway to exert antioxidant effects[22, 56], carrying miR-24-3p for anti-apoptosis and promoting neural repair [23], and promoting angiogenesis through methods like engineered overexpression of HIF-1α [11, 12]. In immunomodulation, it promotes macrophage polarization towards the anti-inflammatory M2 phenotype [16]. Additionally, BMSC-Exos also demonstrate anti-fibrotic potential, inhibiting fibroblast activation and collagen/α-SMA expression, and regulating the Type I/III collagen ratio to improve tissue mechanical properties during anterior cruciate ligament repair [40, 41]. Although these pro-healing mechanisms collectively create a microenvironment unfavorable for pathological scar formation, direct studies specifically focused on the preventive efficacy of BMSC-Exos in skin scar models, especially head-to-head comparative evidence with UCB- Exos or ADSC-Exos, are still relatively lacking. Its anti-scar potential is more inferred from its multiple functional aspects rather than being proven in specific cutaneous scar settings. Therefore, more targeted research is needed to clarify its comparative advantages in clinical scar management.

#### Adipose-derived MSC exosomes (ADSC-Exos)

ADSC-Exos demonstrate strong potential in tissue repair, especially in corneal and skin wound healing, due to their rich bioactive substances. Their functions are diverse, including achieving significant antioxidant and anti-apoptotic effects by regulating the Bax/Bcl2 ratio to protect corneal and skin cells [57], promoting the proliferation and migration of keratinocytes and fibroblasts [58, 59], and exerting anti-inflammatory effects by regulating macrophage polarization [60]. Regarding inhibition of scar formation, ADSC-Exos possess direct evidence: in corneal injury models, it can inhibit the expression of the myofibroblast marker α-SMA and effectively prevent scar formation. Simultaneously, its carried miR-181a-5p can inhibit TGF-β1-induced collagen over-synthesis by targeting Smad2, thereby exerting anti-fibrotic effects. These studies demonstrate good direct evidence of ADSC-Exos in corneal and oral mucosal fibrosis models, indicating its strong translational prospects in skin scar treatment [61] [36].

#### Umbilical cord (Wharton's Jelly) MSC exosomes (UC-Exos)

**Fig. 2:**
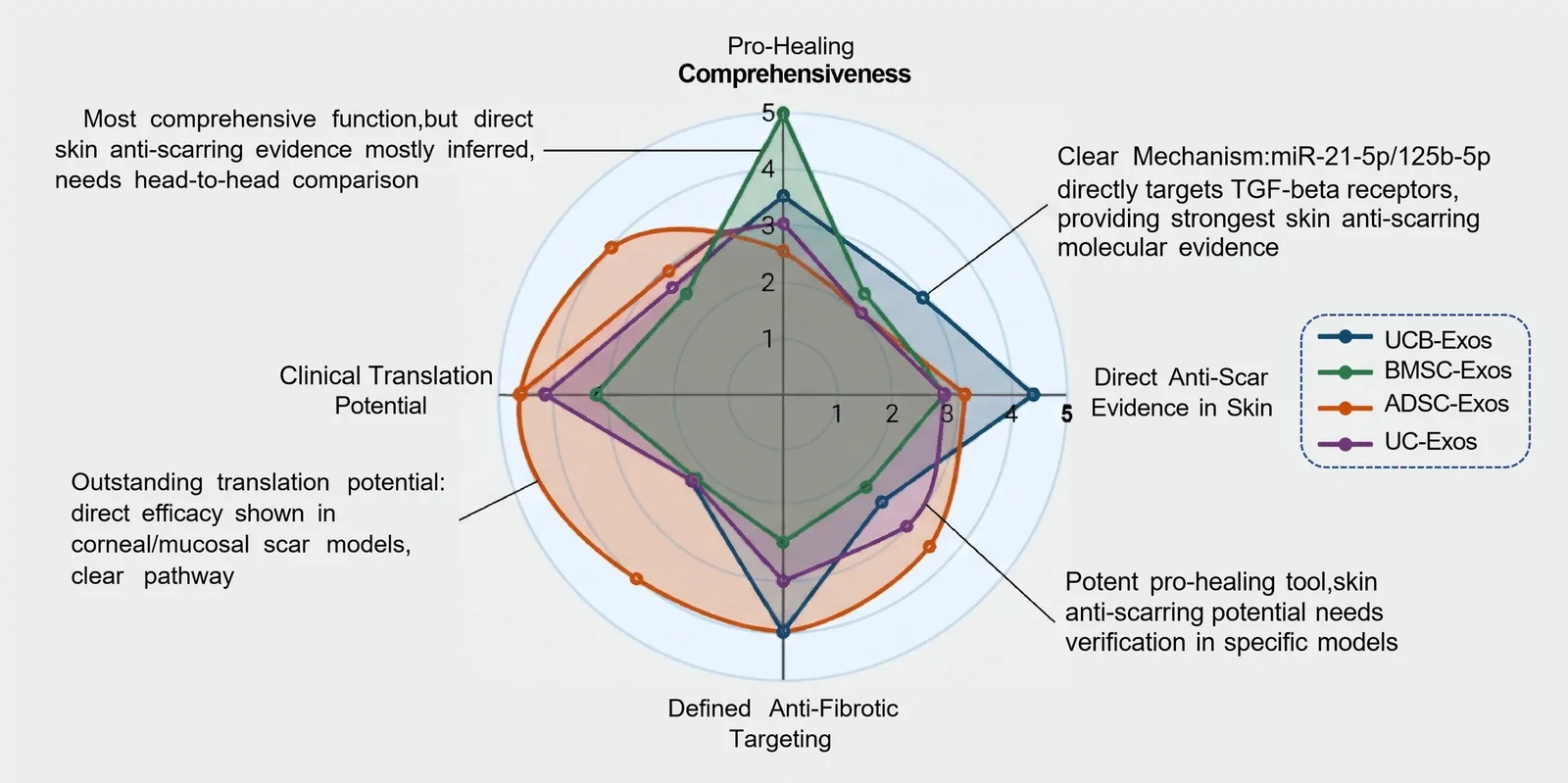
Design scheme for a comparative diagram illustrating functional spectra and anti-scar evidence strength of MSC-exos from different sources

UC-Exos are comprehensive in promoting wound healing. It is rich in fibrinogen, effectively promoting the growth and migration of keratinocytes [62], possesses potent anti-inflammatory capabilities, such as reversing the pro-inflammatory state of chondrocytes in osteoarthritis models [18], and promotes angiogenesis by delivering molecules like angiopoietin-2 [63], thereby accelerating diabetic wound healing [64, 65]. Furthermore, UC-MSC-Exos also exert antioxidant effects in the skin via the miR-200a-3p/Keap1/Nrf2 pathway [56]. Regarding anti-scar potential, exosomes from 3D-cultured UC-MSCs carry let-7i-5p, which targets TGFBR1 to inhibit fibroblast activation in pulmonary fibrosis models [38]. Although evidence for its pro-healing effects is ample, its anti-fibrotic evidence mainly originates from non-skin organ models. Therefore, UC-MSC-Exos are considered a powerful pro-repair tool, but their direct inhibitory effects in specific skin scar formation still require further research for confirmation (Fig. 2, Table 1).

**Table 1:** Comparative summary of MSC-exosomes from different sources

Exosome source	Key healing functions	Key anti-scar evidence/mechanism	Signature molecules
UCB-MSC	Promote wound closure, appendage regeneration	**Direct inhibition** of TGF-β receptors via miR-21-5p/125b-5p, blocks myofibroblast differentiation in skin	miR-21-5p, miR-125b-5p
BMSC	Antioxidant (Nrf2), anti-apoptotic, pro-angiogenic, anti-inflammatory	Inhibits fibroblast activation & collagen/α-SMA in fibrosis models; modulates collagen I/III ratio. Direct skin scar evidence limited	miR-24-3p
ADSC	Promotes keratinocyte/fibroblast activity, antioxidant/anti-apoptotic, pro-angiogenic	Inhibits α-SMA & scarring in cornea; anti-fibrotic via miR-181a-5p/Smad2. Strong translational promise	miR-181a-5p, miR-22-3p
UC-MSC	Promotes keratinocyte migration, anti-inflammatory, pro-angiogenic, accelerates diabetic healing	Carries let-7i-5p targeting TGFBR1 (lung fibrosis model). Antioxidant via miR-200a-3p/Nrf2 in skin	Ang-2, let-7i-5p, miR-200a-3p

### Clinical applications and challenges

#### Standardization and manufacturing

The clinical translation of MSC-Exos are fundamentally constrained by the need for standardized, large-scale manufacturing. Traditional isolation methods like ultracentrifugation suffer from low yield and purity, leading to significant batch-to-batch variability that hinders reproducible therapeutic outcomes [66, 67]. Adherence to Good Manufacturing Practice (GMP) standards and characterization guidelines, such as those proposed by the International Society for Extracellular Vesicles (MISEV), is essential [68–71]. Future advancements hinge on developing scalable technologies, like 3D cell culture and novel isolation platforms, to ensure consistent production of high-quality exosomes [72, 73].

####  Potency assays and quality control

Moving beyond basic physical characterization (size, concentration, marker proteins) is critical. There is an urgent need to establish robust, biologically relevant potency assays that directly correlate with clinical efficacy. Examples include quantifying the ability of exosomes to inhibit TGF-β-induced myofibroblast differentiation or to promote endothelial tube formation in standardized assays. Such functional quality controls are indispensable for ensuring batch consistency and predicting in vivo therapeutic performance [74–77].

#### Delivery and targeting strategies

Optimizing delivery is paramount to enhance efficacy and minimize off-target effects. While topical application, injectable hydrogels, and scaffold-based systems offer various routes [78–80], achieving precise targeting remains a challenge. Surface engineering of exosomes, for instance by conjugating targeting peptides like RGD, is a promising strategy to improve their homing and retention at specific wound sites, thereby enhancing therapeutic precision and reducing required doses [72, 81, 82].

#### Safety and regulatory landscape

Although MSC-Exos are generally considered safe with low immunogenicity, comprehensive long-term safety data are lacking. Key concerns include their in vivo biodistribution, potential for off-target immune modulation, and theoretical tumorigenicity risks [83–86]. The regulatory pathway for exosomes as therapeutics is still evolving, requiring clear dialogue with agencies like the FDA and EMA. Rigorous preclinical and clinical studies are needed to build a robust safety profile and navigate the complex regulatory framework.

#### Functional heterogeneity and context-dependency

A profound and often overlooked challenge is the context-dependent functionality of MSC-Exos. Their effects are not universally pro-healing; they can vary dramatically based on the parent cell source, preconditioning, and the specific disease microenvironment. A pivotal study on post-myocardial infarction scarring revealed that a specific subpopulation of PDPN + MSC-derived exosomes could exacerbate fibrosis by promoting amyloid deposition, highlighting their potential dual role [19]. This inherent heterogeneity cautions against over-generalization and underscores the necessity for rigorous, disease-specific preclinical validation to ensure predictable clinical outcomes.

**Fig. 3:**
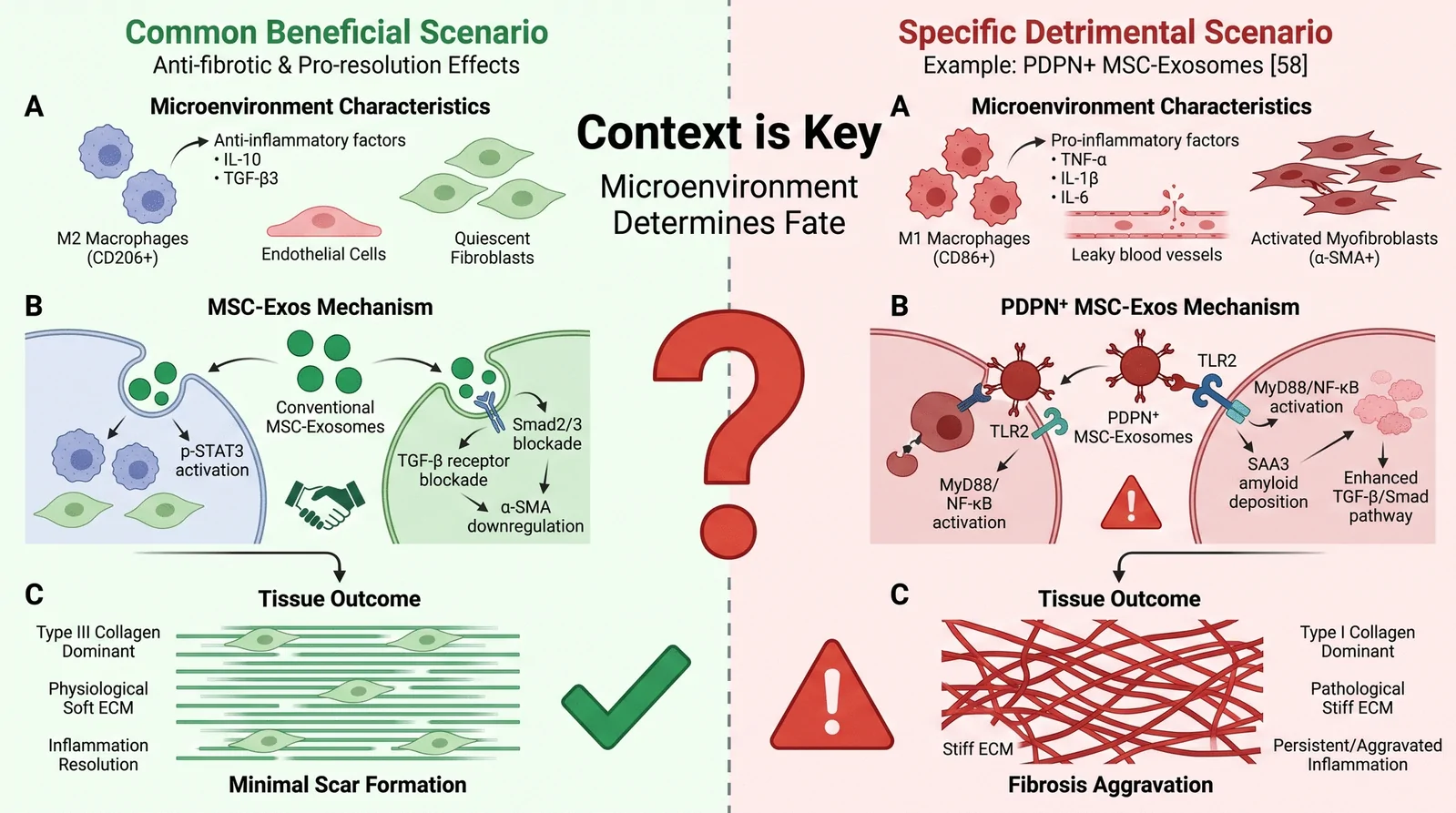
MSC-exos function’s double-edged sword and context-dependent warning diagram

In summary, MSC-derived exosomes face multiple challenges in clinical translation, including standardized production, potency characterization, targeted delivery, and safety assessment. Moreover, the "context-dependent" and "heterogeneous" nature of their functions represents a core biological characteristic that must be thoroughly understood prior to their application. To provide a more intuitive overview of the "double-edged sword" effects of MSC-derived exosomes in wound healing and scar prevention, as well as the key bottlenecks in their clinical application, we have summarized these critical mechanisms and challenges in Fig. 3.

### Advanced biomaterial-based delivery strategies for MSC-Exos

Although MSC-Exos demonstrate immense potential in wound healing and scar prevention, their naked application faces significant challenges that severely limit therapeutic efficacy, including rapid clearance, short local retention time, insufficient targeting, and instability of active components. Given the functional heterogeneity of MSC-Exos from different sources and the context-dependent challenges in their application, achieving targeted and sustained delivery becomes paramount. Combining MSC-Exos with advanced biomaterial carriers represents a key strategy to overcome these hurdles and enable their clinical translation. These material systems primarily aim to address three core issues: enhanced stability, controlled release, and targeted delivery.

#### Types and functions of carrier systems

**Fig. 4:**
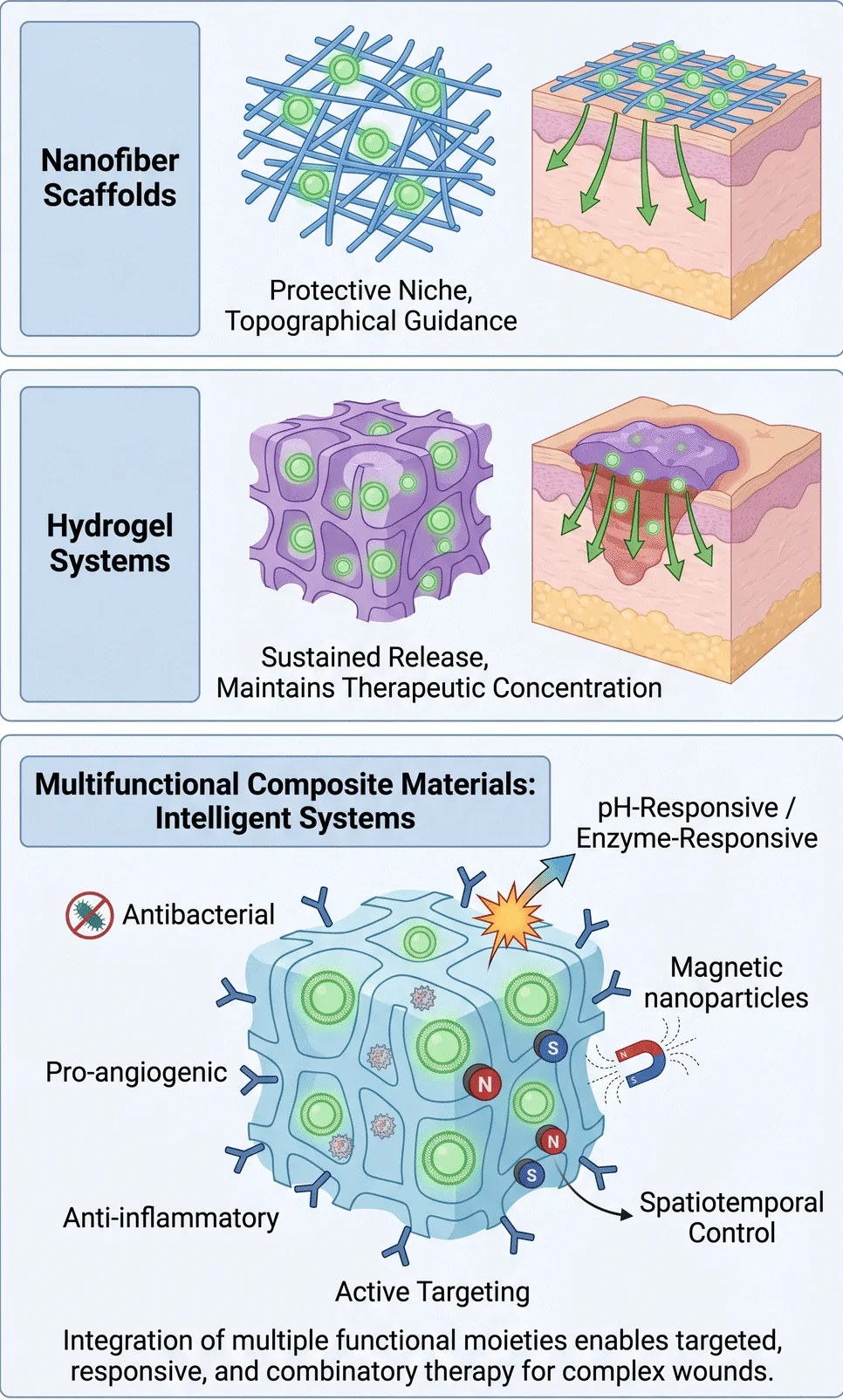
Schematic of advanced biomaterials as exosome delivery systems

Carrier systems for MSC-Exos are primarily designed to address the critical challenges of rapid clearance and unstable delivery. As shown in Fig. 4, they can be broadly categorized into three main types, each with distinct structural and functional advantages: Nanofiber scaffolds (e.g., electrospun nanofibers) are characterized by high porosity and an ECM-mimicking structure. Their core advantages include providing a protective niche for exosomes and topographically guiding cell behavior. For instance, MSC-Exos loaded in nanofiber patches not only leverage the material's inherent antibacterial and exudate-management properties but also synergistically enhance biological activity, demonstrating efficacy in promoting scarless healing in acne wounds [87, 88]. Hydrogel systems, based on materials like hyaluronic acid, chitosan, and gelatin, enable the sustained release of exosomes, maintaining effective therapeutic concentrations at the wound site. For example, a recombinant collagen/carboxymethyl chitosan composite hydrogel loaded with human umbilical cord MSC-Exos achieves long-term exosome release through its porous structure. While promoting fibroblast proliferation, alleviating inflammation, and enhancing angiogenesis, it also effectively reduces scar formation [89]. Photopolymerized methacrylated gelatin hydrogels have also been proven to significantly accelerate diabetic wound healing [82]. Multifunctional composite materials further endow carrier systems with intelligent properties by integrating various functional components, such as bioactive nanoparticles, targeting peptides, and magnetic particles. For instance, multifunctional composite hydrogels loaded with exosomes can simultaneously exert antibacterial, pro-angiogenic, and anti-inflammatory effects to manage complex wounds [90]. Combining exosomes with magnetic nanoparticles allows for spatiotemporally precise targeting and regulation via an external magnetic field, offering a novel approach for intelligent therapy.

#### Clinical translation challenges and future directions

Despite the promising prospects of biomaterial carrier systems, the clinical translation of their combination with MSC-Exos faces a series of challenges. Future development should focus on in-depth integration and optimization in the following directions. Precision engineering of the carrier-exosome interface requires a deep understanding and optimization of the interaction between exosomes and carrier materials. The goal is to improve loading efficiency and achieve intelligent controlled release responsive to the wound microenvironment (e.g., pH, enzymes) while ensuring the biological activity of exosomes is not compromised. Integration of scalable manufacturing and quality control necessitates that the large-scale, standardized production of biomaterial carriers be seamlessly integrated with the GMP-compliant production process for exosomes. A comprehensive set of quality control standards encompassing physical characteristics, loading efficiency, release kinetics, and in vitro biological activity needs to be established. Targeting and personalized delivery can be further enhanced by surface modification of carrier materials (e.g., conjugation with targeting peptides like RGD) or designing flexible patches that conform to complex wound surfaces, thereby increasing their accumulation and retention at specific wound sites for precise treatment [72, 81]. In the future, developing personalized carrier systems tailored to wounds of different etiologies (e.g., diabetic ulcers, burns, surgical incisions) and locations will be an important direction. Strengthening preclinical validation and clinical trial design urgently calls for the systematic evaluation of the long-term safety and efficacy of drug-loaded systems in more clinically relevant, complex animal models, such as chronic wound models with comorbidities. Building on this, promoting the design of rigorous clinical trials that stratify outcomes based on administration route, dosage, treatment course, and efficacy against different types of wounds/scars is a crucial step in confirming their clinical value [87, 91].

In summary, the combination of biomaterials with MSC-Exos provides a powerful tool to address the clinical application bottlenecks of the latter. Through interdisciplinary collaboration, in-depth optimization of carrier design and production processes, and the advancement of robust clinical research, this combined therapeutic strategy holds promise for a successful translation from the laboratory to the clinic. It aims to provide patients with more efficient and safer solutions for wound repair and scar management (Fig. 4).

## Conclusion

MSC-derived exosomes represent a transformative, cell-free therapeutic strategy with immense potential for skin wound healing and scar prevention. This review underscores that their efficacy stems from a multi-mechanistic ability to orchestrate the healing process—promoting cell proliferation, modulating inflammation, protecting against oxidative stress, and stimulating regulated angiogenesis. Most significantly, certain MSC-Exos, particularly those from umbilical cord blood, can directly target the core drivers of fibrosis, such as the TGF-β/Smad pathway, to inhibit myofibroblast differentiation and aberrant collagen remodeling.

However, the path to the clinic is not without hurdles. Critical unresolved questions remain: Which MSC source provides the optimal exosome profile for specific wound types? What is the definitive therapeutic time window and dosage? How can we reliably track exosome fate in vivo? The paradoxical, context-dependent functions of exosomes, exemplified by the fibrosis-promoting PDPN + MSC-Exos [19], serve as a crucial reminder of the complexity of this biological system and the need for meticulous characterization.

Future research priorities should therefore focus on: (1) Conducting head-to-head comparative studies of exosomes from different sources in standardized skin scar models. (2) Engineering and priming strategies to enhance desirable functions (e.g., anti-fibrotic miRNA loading) and ensure safety. (3) Developing advanced biomaterial carriers for controlled, targeted, and sustained local delivery, and (4) Initiating rigorously designed clinical trials that address standardization, potency, and long-term outcomes. Only through such interdisciplinary and focused efforts can the remarkable promise of MSC-Exos be fully harnessed to achieve scar-minimized, regenerative healing for patients worldwide.

## Data Availability

The data presented in this study are available on request from all authors.
